# Exploring urban mental health using mobile EEG – a systematic review

**DOI:** 10.1371/journal.pmen.0000203

**Published:** 2025-04-04

**Authors:** Ben Senkler, Sophie Klara Schellack, Toivo Glatz, Julius Freymueller, Claudia Hornberg, Timothy Mc Call

**Affiliations:** 1 Sustainable Environmental Health Sciences, Medical School OWL, Bielefeld University, Bielefeld, North Rhine Westfalia, Germany; 2 Berlin School of Public Health, collaborative initiative of Charité - Universitätsmedizin Berlin, Alice Salomon University of Applied Sciences (ASH Berlin) and Technical University Berlin, Berlin, Germany; 3 Institute of Public Health, Charité – Universitätsmedizin Berlin, corporate member of Freie Universität Berlin and Humboldt-Universität zu Berlin, Germany; 4 Working Group 7 Environment and Health, School of Public Health, Bielefeld University, Bielefeld, North Rhine Westfalia, Germany; Sigmund Freud University Vienna, AUSTRIA

## Abstract

Given the ongoing trend of urbanization and the increased prevalence of specific mental disorders in urban settings, there is a need to better understand the link between urban living and mental health. Recent advances in urban mental health research have leveraged mobile electroencephalography to explore how brain electrical signals are influenced by urban stressors and resources. This study aims to synthesize the evidence from mobile electroencephalography measurements in the context of urban mental health. A systematic literature research was conducted in the databases PubMed/MEDLINE, Embase, PsycINFO and CINAHL in September 2023. The present review includes primary studies that used in-situ electroencephalography in real urban environments published since 2013. Four independent reviewers conducted the screening, while two researchers performed data extraction using Microsoft Excel and assessed risk of bias using the Effective Public Healthcare Panacea Project Quality Assessment Tool. The review has been pre-registered with the International Prospective Register of Systematic Review (PROSPERO) under the registration number CRD42023471636. Fifteen studies were identified, primarily examining power in alpha, beta, and theta frequencies in urban areas compared to less urbanized environments. Study findings exhibited significant heterogeneity; while some studies noted heightened brain activity in urban environments, others observed reductions compared to less urbanized or greener regions. Notably, certain demographic cohorts, such as adolescents, have been understudied. Moreover, descriptions of exposures were often inadequate for ensuring replicability, and gender considerations were seldom integrated into analyses. This systematic review provides insights into an emerging field of research which appears to be suffering from small sample sizes and a lack of methodological transparency and consistency. Interpretation of the seemingly contradictory results requires future studies to be more rigorous in documenting urban exposures and choice of brain components under investigation.

## 1. Introduction

As the spatial distribution of the world’s population shifts, with urban areas expanding relative to rural areas, exploring the complex interplay between urban environments and human health is becoming an increasingly important topic for public health research [1,2]. The United Nations predicts that 68% of the world’s population will live in cities by 2050. Similarly, the World Health Organization stresses the importance of equitable, sustainable and healthy urban environments, e.g., by providing universal access to safe, inclusive and accessible green and public spaces, and refers to the inclusion of this objective in the Sustainable Development Goals [3].

Besides a variety of health-promoting aspects linked to urban life, such as a more concentrated range of educational opportunities and support, improved medical services and a diverse cultural offering, environmental disadvantages are also evident [4]. Epidemiological studies show that many stress-related mental disorders occur more frequently in cities than in rural regions [5,6]. For example, higher prevalences have been reported for conditions such as anxiety disorders [7], mood disorders [8] and especially psychotic disorders such as schizophrenia [9]. Adolescence or growing up in an urban environment may be particularly associated with the development of schizophrenia. Pedersen and Mortensen [10] even describe the association as a dose-response relationship for growing up in urban areas in Denmark in the 1950s through 1990s, stating that the more time spent in an urban environment, the higher the risk of schizophrenia in adulthood. Another study was also able to show that a higher density of housing units per km^2^ in the neighborhood and a low density of green spaces are each independently associated with more psychotic experiences [11]. However, the underlying reasons for these observations are not yet fully understood [4]. While some of the studies suggest a causal relationship between living and growing up in cities and mental illness, others point to selective processes that are responsible for the emergence of mental illness. A relevant environmental factor for health outcomes in urban populations is the natural environment, in particular green spaces and green elements. These are defined in a number of ways across disciplines, which can potentially decrease the comparability of studies and findings [12]. While Taylor & Hochuli [13] identified two potential definitions for green space, the authors emphasize that there is no general definition for green space. They highlight the necessity to outline the definition of green space used in the particular study.

Various theories are used to approach the differences in the effects between the built and natural environment. The Attention Restoration Theory, for example, assumes that sensory stimuli of natural environments can reduce stress and mental fatigue compared to urban environments [14,15]. Kaplan & Kaplan [15] state that, the stimuli of these environments could require less directed attention and could therefore be processed more effortlessly. Consequently, there could be a difference between “top down attention”, where a higher level of cognitive effort is required, such as at busy road crossings, as opposed to “bottom up” attention, where natural environments attract attention through the fascination of the stimuli [16,17]. Another leading theory dealing with explanations for natural and urban effects on stress is the “Stress Reduction Theory” (SRT) proposed by Ulrich et al. [18]. The SRT states that the psychological stress response is attributed to many years of evolution in nature, which has resulted in humans having an innate preference for natural rather than artificial environments and thus decrease mental stress through natural settings [19]. However, the SRT does not further describe any pathways that explain the effects. Nevertheless, research into the underlying mechanisms is essential, as it could enable accurate evidence-based measures for the health-promoting design of urban environments [20].

In response to a better understanding of the mechanisms of urban environments on mental health and cognition, there are several different disciplines and approaches, with the neurosciences coming to the fore in recent years. As a result, methods such as magnetic resonance imaging (MRI) for investigating morphological brain changes and electroencephalography (EEG) for assessing neuronal responses are being used in an attempt to relate these changes to urban life [21]. However, existing EEG studies measuring brain electrical signals are often conducted in the laboratory, with subjects only exposed to image-based slideshows or virtual reality videos of urban environments for the assessments [22,23]. Alternatively, methodological approaches that can be applied directly in the environment and in real time, meaning mobile methods, are receiving increasing attention [24]. This also applies to “mobile EEG” as a relatively new method for observing the brain in natural and urban environments. Mobile EEG is considered relatively inexpensive, and allows a higher degree of movement during measurement [25]. EEG measurements can then be utilized to draw inferences about mental states and certain emotions such as excitement, alertness and anger [26]. Similarly, possible cognitive parameters such as attention, working memory or cognitive load can be assessed [24,25]. There are also studies that discuss specific EEG signals as biomarkers for manifesting mental illnesses, such as anxiety disorders [27] or depressive disorders [28,29]. Therefore, employing mobile EEG technology in urban settings could enable the investigation of neuronal processes, spatial perception, and cognition [30]. Consequently, comparing power differences within different frequency bands (e.g., alpha, beta, gamma), observing measures of brain functional connectivity, or measuring event-related potentials may offer valuable insights into the influence of urban environments on the mental well-being of residents. Although mobile EEG is described as a promising tool for understanding urban behavior and exploring the psychological impact on individuals, the evidence for this new approach in the context of mental health in urban areas has not yet been provided in a systematic way. Gramann [31], in his critical review, describes a strong trend in neuro-urbanist studies, coupled with an increase in mobile EEG research conducted in real-world environments. Particularly due to the availability of affordable, consumer-grade software, recent years have seen a significant rise in urban EEG studies. However, these studies often exhibit methodological limitations and errors that constrain the reliability of their findings. Against this background, a systematic investigation appears necessary to thoroughly analyze the current body of research and critically assess its validity.

To address the identified research gap, the existing literature on this topic will be systematically reviewed to explore the following research question: What are the results of studies investigating the relationship between exposure to urban environments and brain signals measured by mobile EEG published between 2013 to September 2023? As an additional question incorporated later in the process, the mental health parameters considered by the included studies will also be addressed.

## 2. Methods

The reporting of this review is based on the Preferred Reporting Items for Systematic Reviews and Meta-Analyses (PRISMA) 2020 to comply with the criteria of good scientific practice [32] (S1 Table). The systematic literature search is being conducted using the medical and psychological databases CINAHL, PubMed/MEDLINE, PsycInfo, and Embase. The search was last updated on September 27^th^, 2023. Additionally, the study protocol has been pre-registered with the international prospective register of Systematic Review (PROSPERO) under the registration number CRD42023471636 [33] and in the Supplementary Material (S1 Text). The respective search strategies for each database can be found in the Supplementary Material (S2 Table). After identifying the database records, the studies are further processed using the tool Rayyan [34]. This involves automatic detection of duplicates, which are checked by one reviewer (BS). First the screening of titles/abstracts is done in Rayyan (BS, SKS, JF, TMc). The screening of full-texts is conducted in Microsoft Excel by two independent reviewers (BS, SKS). Studies are selected according to strict inclusion and exclusion criteria, which are listed in Table 1. During the full text screening, the literature references of the selected studies are checked for possible further studies that could match the inclusion/exclusion criteria. Any disagreements about the inclusion or exclusion of individual studies are resolved by a third reviewer (TMc). The individual screening decisions can be accessed in Supplementary Material (S3 Table). A broad data extraction form is designed to provide a comprehensive overview of the study, encompassing the variables year of measurement, country/region, population description, analyzed participants (number, gender/sex), drop outs/not analyzed, mean age (age in years, range), study design, within-subject/between-subject, randomization, compared exposure, urban exposure (author stated), tools for identification of environmental features, time under measurement, steady/moving exposure, EEG-outcome, electrode placement, measured brainwave components, main results/direction of effect, mental health interest, interpretation (author stated) and further measures of mental health. Finally, the data extraction process also examined whether the authors accounted for any sex and gender differences that could influence the results in their design or analysis of measurements. The data extraction is conducted with 100% overlap, with both extractors (BS, SKS) extracting all data into an Excel spreadsheet and subsequently checking for discrepancies. Disagreements in the extraction process were discussed between the two researchers (BS, SKS) and, in case of disagreement, resolved by a third reviewer (TMc).

**Table 1:** Inclusion/exclusion criteria.

Inclusion criteria	Exclusion criteria
1) Primary studies 2) At least one outcome measured by mobile/in-situ EEG separately reported 3) Exposure includes at least one urban phenomenon (e.g., walking in an urban green space; being near a traffic road; etc.) 4) Written in English or German language	1) Reviews or meta-analyses 2) No urban exposure/urban exposure not as focus of the study 3) Virtual/augmented reality for urban exposure 4) Studies including other than human subjects (e.g., animals) 5) Studies with publication date before 2013

To evaluate the study quality of the selected studies, a quality assessment is conducted using the Effective Public Healthcare Panacea Project (EPHPP) Quality Assessment Tool [35]. The entire assessment is also carried out independently by two researchers (BS, SKS) and then compared. Discrepancies are initially resolved in a discussion between the two researchers; if no agreement could be reached, a third researcher is consulted (TMc). Data is narratively reported using the extraction table, facilitating comprehensive presentation. In both data extraction and synthesis processes, any missing or unreported data is explicitly identified and documented.

## 3. Results

**Fig. 1:**
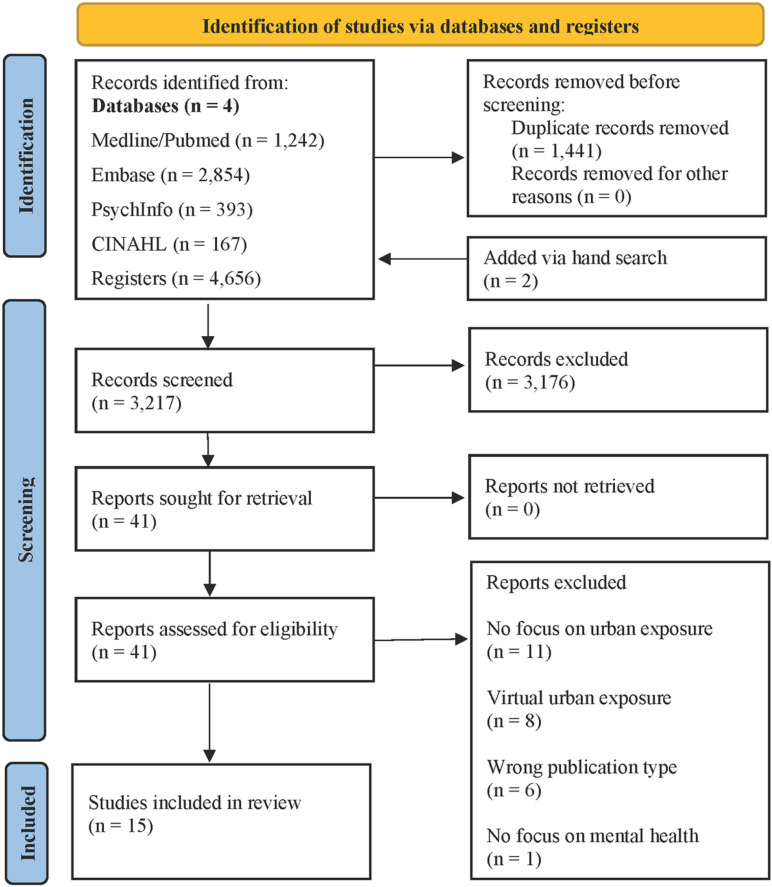
Flowchart of study selection progress.

The database search identified 4,656 publications, of which 1,441 duplicates were removed. A total of 3,215 records were screened in the title and abstract screening, resulting in an exclusion of 3,176 records. After the screening process, two studies were added by hand search looking at citation lists. A total of 41 studies were further assessed in the full-text screening process. After the exclusion of 16 studies in the full-text screening, 15 studies were included in the review. Further details of the screening process are provided in Fig 1. The supplementary S4 Table shows the characteristics and the results of the included publications.

### 3.1. Participants

Within the 15 included studies, the number of participants analyzed ranged from 8 [36] to a maximum of 95 participants [17,37]. Eleven studies contained mixed-gender participant populations [14,36,38–46]. In addition, two interventions included only female participants [47,48], while no studies incorporated only male participants. A further two studies did not describe the gender distribution [17,37]. The youngest mean age observed among participants was 19 years [40], while the oldest mean age recorded was 76 years [17,37]. In absolute terms, the age range is 17 [47] to 92 [17,37] years. Six studies specifically targeted young university students [38–41,47,48], while four studies focused on older adults, defined as those over 65 years of age [17,36,37,46]. Almost all studies (n = 14) either limited their samples to neurotypical individuals or did not specify any additional exclusion criteria. However, one study explicitly included participants with depression [43]. Dropout rates were reported in only five studies [14,36,41,45,47], while the remaining ten studies did not document dropout rates. The 15 studies were conducted in seven different countries. Five studies were performed in Europe, all of which were based in the United Kingdom [17,36–38,44]. A further five trials were carried out in eastern and south east Asia [40,42,43,46,48]. Two studies were conducted in North America [41,45] and one in Australia [14]. Finally, two studies did not specify the country or region in which they were performed [39,47]. Table 2 summarizes the characteristics of participants from the studies included in the analysis.

**Table 2:** Characteristics of the study participants.

Author, Year	Country	Number of participants (% female)	Mean age; (range)
Al-Barrak et al., 2017 [47]	(N/A)	40 (100%)	21.5; (17-30)
Aspinall et al., 2015 [38]	United Kingdom	12 (33.33%)	30.1; (N/A)
Baumann & Brooks-Cederqvist, 2023 [14]	Australia	28 (71.43%)	27.20; (19-63)
Chen et al., 2016 [39]	(N/A)	32 (50%)	20.6; (N/A)
Elsadek et al., 2019 [48]	China	25 (100%)	23; (N/A)
Hassan et al., 2018 [40]	China	60 (50%)	19.6; (N/A)
Hopman et al., 2020 [41]	United States of America	29 (65.52%)	25.48; (N/A)
Neale et al., 2017 [17]	United Kingdom	95 (N/A)	76.55; (65-92)
Neale et al., 2020 [37]	United Kingdom	95 (N/A)	76.55; (65-92)
Olszewska-Guizzo et al., 2020 [42]	Singapore	22 (59.09%)	32.9; (N/A)
Olszewska-Guizzo et al., 2022 [43]	Singapore	92 (59.78%) (discrepancy between text and table)	36.62; (21-74)
Reeves et al., 2019 [44]	United Kingdom	34 (61.76%)	41; (N/A)
Scanlon et al., 2020 [45]	Canada	10 (40%)	23.4; (20-31)
Tilley et al., 2017 [36]	United Kingdom	8 (62.50%)	75.75; (67-86)
Torku et al., 2022 [46]	Hong Kong	10 (70%)	68.0; (65-75)

N/A = not applicable or not available

### 3.2. Study design

All studies involved an experimental intervention. While five studies utilized a within-subject design [38,41,44,46,47], another five studies divided the groups, thus employing a between-subjects design [17,37,39,40,43] and a further set of five studies used elements of both designs [14,36,42,45,48]. Within these study designs, various methods of participant randomization were utilized. Specifically, four studies randomized participants into distinct exposure groups [17,37,39,43], whereas six studies randomized the participants order of exposures to control for site-order effects [14,36,40,42,45,48]. Notably, five studies did not report any randomization procedure [38,41,44,46,47].

### 3.3. Urban exposure vs. Compared exposure

The following section provides an overview of the different exposures examined and categorize them in the included studies according to duration, type of environment and its operationalization, as well as the type of movement through the respective sites. In 14 studies, the level of greenery was the main factor that varied between urban and control exposure conditions [17,36–48]. For example, control conditions often included settings with higher levels of greenery, such as parks compared to more urbanized/built areas, or nature walks compared to urban exposures. Only one study compared urbanity based on building density [14]. Similarly, there was variation in the methods of exposure among the studies. Eight studies used a moving exposure approach [17,36–38,40,45–47], involving activities such as walking or cycling through the designated area. Conversely, seven studies opted for a stationary exposure method, where participants remained seated in front of an urban environment or the comparator exposure [14,39,41–44,48]. The duration of the measured exposure varied between the studies. The shortest measurement duration observed was only 3 minutes (1 minute per site) [42], while the longest measurement duration was 30 minutes (15 minutes per site) [40]. Two studies did not report the duration of measurement [36,46]. Additionally, different methods were used to operationalize the diverse urban and compared exposures. For example, Tilley et al. [36] employed Geographic Information Systems to categorize quartiles ranging from urban green to urban busy, providing a detailed description of green and urban features. Olszewska-Guizzo et al. [42,43] analyzed selected viewpoints with the “Contemplative Landscape Model” using four landscape architecture experts in a blinded manner. This approach allowed for the calculation of an average score for each site, reflecting the quality of surrounding landscape characteristics. Torku et al. [46] utilized a specialized tool for the city of Hong Kong, capturing environmental conditions like aesthetic appeal, safety, and functionality. Chen et al. [39] operationalized the environment through professional photographs of viewpoints, analyzing the distribution of green spaces and built-up areas. In nine other studies, no additional measures were taken to further describe the environments [17,37,38,40,41,44,45,47,48].

### 3.4. Main outcome

The 15 selected studies investigated brain activity within various frequency bands and brain regions as measured by mobile EEG. Detailed information on electrode placements can be found in the Supplementary material (S4 Table). In the following chapters, the results of the various EEG paradigms, such as spectral power analysis, affective computing and other measures, are reported. In our selected studies, frequency measures were primarily used, with only one study utilizing ERP or other time-domain features. This preference likely stems from the lack of controlled stimulus onset presentations, which are generally required for time-domain analysis. However, some studies employed blink- or fixation-based ERP paradigms in real-world settings [45]. Frequency-based measures are more commonly used because they capture ongoing brain activity without requiring precise timing, making them particularly well-suited for dynamic settings. In contrast, ERP measures, which depend on specific timing, are less frequently employed but remain feasible when alternative techniques are applied. Futhermore underlying processes, which are reportedly reflected by the measured EEG paradigm are extracted and synthesized.

#### 3.4.1 Power spectrum analysis.

The subsequent section gives an analytical description the studies regarding power in the frequency bands Delta, Theta, Alpha, Beta, Gamma. Table 3 lists the individual frequency bands, consistently comparing built/urbanized environments with greener/less urbanized areas.

**Table 3:** Analysis of Power Spectrum by Degree of Urbanization.

Power Spectrum Analysis			
	**Higher power reported for urbanized areas compared to less urbanized**	**Lower power reported for urbanized areas compared to less urbanized areas**	**no differences in power found between urbanized and less urbanized areas**
Delta (<4Hz)		Chen et al. [39]	Reeves et al. [44]
Theta (4-8Hz)	Hopman et al. [41]	Chen et al. [39] Baumann & Brooks-Cederqvist [14]	Torku et al.[46] Reeves et al. [44]
Alpha (8-13Hz)	Neale et al. [37] Hopman et al. [41]	Chen et al. [39] Hassan et al.[40] Elsadek et al [48]	Baumann & Brooks-Cederqvist [14] Reeves et al. [44] Torku et al. [43]
Beta (13-30Hz)	Baumann & Brooks-Cederqvist [14] Neale et al. [17]	Chen et al. [39] Hassan et al.[40] Reeves et al. [44]	Torku et al.[46]
Gamma (>30Hz)			Chen et al. [39] Torku et al. [46]

##### 3.4.1.1. Alpha

The most commonly assessed frequency band was alpha, with ten studies reporting on it [14,37,39–44,46,48]. When comparing general differences in spectral power according to environmental exposure, there are two studies that report an increase in alpha power in urban areas compared to less urbanized areas [37,41], while three studies found a decrease in urban areas [39,40,48], another three studies were unable to identify significant results [14,44,46]. Additionally, two studies deviated from focusing solely on absolute spectral power and instead examined Frontal Alpha Asymmetry (FAA) as a parameter [42,43]. Of the studies examining alpha activity, five did not report on the topographical distribution of these effects [14,37,39,40,46], whereas the other five did provide information on their localization [41–44,48].

When examining the different environmental conditions of the studies that reported an increase in urban areas, Hopman et al. [41] observed higher posterior alpha power and mid-frontal alpha power when exposed to campus buildings as an urban exposure, while Neale et al [37] observed an increase in a busy urban environment, described as an area with a predominance of buildings, mainly residential area with some front gardens, and paved areas compared to an urban quiet environment, which was referred to as sections with a predominance of buildings, paved areas and a commercial street frontage, which attracts high pedestrian and vehicular traffic. However, in their interventional studies, no statistically significant differences in alpha power between an urban quiet environment and an urban green environment, depicted as a region with a predominance of vegetated and unbuilt surfaces, or between the urban- busy and green urban areas were observed.

In contrast, the three other studies that found an increase in the compared, less urbanized environments [39,40,48] carried out their experiments in different environments. Elsadek et al. [48] found that the left prefrontal lobe (AF3) exhibited higher alpha relative power when facing the green facade compared to the building wall. Similarly, the right prefrontal lobe (AF4) showed higher alpha relative power when viewing the green façade compared to the building wall. In the left occipital lobe (O1), there was also higher alpha relative power when viewing the green façade compared to the building wall. Likewise, the right occipital lobe (O2) showed greater relative alpha power when viewing the green façade compared to the building wall. Hassan et al. [40] compared a walk in a bamboo forest environment with a walk in an urban area with traditional buildings. Chen et al [39] identified an increase in a wooded campus with 89% visible greenery and 4% paved areas, compared to a traffic island with 8% visible greenery and 56% visible buildings.

In addition, three other studies [14,44,46] were unable to identify significant associations between alpha power and environmental conditions.

Two studies investigated the specific parameter of FAA across different conditions [42,43]. They examined the hemispheric differences in alpha power in the frontal lobe. In the study from Olszewska-Guizzo et al [43] in 2022, no difference in FAA was found between urban and green control sites within the overall sample. However, when the sample was separated into healthy and clinically depressed individuals, distinct patterns emerged. Specifically, during exposure to a “therapeutic garden”, the control group exhibited left-sided FAA, whereas the clinical sample indicated right-sided FAA. The exposure to a “busy downtown” environment revealed right-sided FAA in the healthy group and left-sided FAA in the clinical sample. In the “residential green” setting, the EEG measurements showed a right-sided FAA in both the healthy group and the clinical group. In another study from Olszewska-Guizzo et al [42] in 2020, the authors found no significant differences between “urban park” compared to “urban street” for FAA and no significant differences between urban park compared to neighborhood green. However, the authors found an increased right-sided FAA when comparing “neighborhood green” and “urban street”.

The authors offer various interpretations within the studies as to the extent to which the changes in alpha band could affect emotional or cognitive domains. Four studies [37,40,44,48] interpret an increase in power across the alpha frequency band as a state of relaxation, meditation or a resting state. For example, Elsadek et al. [48] assumed, being exposed to green facade makes participants feel more wakefully relaxed in contrast to looking at a building wall. Neale et al. [37] suggested that the increase in alpha power may reflect the familiarity of urban elements. They propose that the density of familiar stimuli is higher in urban busy and therefore higher levels of alpha can be observed. Hopman et al. [41] and Elsadek et al. [48] go on to describe that alpha frequencies are also related to attention. They describe that higher levels of alpha power are associated with an increase in internal processing or higher attention. Olszewska-Guizzo et al. [42] pointed out in their interpretation of FAA that a decrease in right frontal lobe compared to left frontal lobe could be associated with positive emotions such as happiness and calmness and the opposite FAA with negative emotions such as fear and sadness. The latter was also found in patients with anxiety disorders and depression. Additionally, Olszewska-Guizzo et al. [43] also refers to the occurrence of left-hemispheric FAA in depressed patients. Three further studies do not provide a direct interpretation of the differences found in alpha band [14,39,46].

##### 3.4.1.2. Beta

An analysis of beta-band activity was conducted across seven studies [14,37,39,40,43,44,46]. In urban environments compared to less urbanized regions, three studies observed a decrease in beta wave power [39,40,44], whereas two studies indicated the contrary [14,17]. In addition, one study found no significant results [46], while another examined the parameter of temporal beta asymmetry (TBA) [43]. Out of the seven studies examining beta power, only one provided information on the topographical localization of the effect [43].

Reeves et al. [44] and Hassan et al. [40] observed an increase in high beta waves in a managed wetland and bamboo forest. Furthermore, Chen et al. [39] also reported a decrease in overall beta band power during exposure to a forested campus with a predominance of greenery. While Reeves et al. [44] and Chen et al. [39] used an urban environment with a busy intersection as a comparison, Hassan et al. [40] exposed participants to an urban area with traditional buildings. In contrast, two studies reported an increase in beta wave power in urban environments compared to less urbanized regions/natural environments [14,17]. The first study identified an increase during exposure to medium-density urban environments compared to low-density environments [14]. Investigating transitions from “urban busy” (predominance of buildings, paved areas, and a commercial street frontage attracting high pedestrian frequency and significant vehicular traffic) to “urban green” (predominance of vegetated and unbuilt surfaces), Neale et al. [17] found no differences in the high beta band but observed an increase in the low beta band in urban busy compared to urban green. Transitions from “urban quiet” (predominance of buildings, some front gardens and paved areas, but these are mainly residential) to “urban busy” showed no significant differences in either high or low beta activity. Furthermore, no statistically significant results were found for transitions between “urban quiet” and “urban green”.

Within the beta band, the EEG marker TBA was analyzed. TBA refers to the hemispheric differences in beta power in the temporal lobe. Olszewska-Guizzo et al. [43] examined TBA across various conditions. Although no differences were observed across sites within the overall sample, distinct patterns emerged when stratifying participants into healthy controls and clinically depressed groups. Specifically, within the “busy downtown” setting, the healthy group showed right-sided TBA, while the clinical sample displayed left-sided TBA. However, these findings did not reach statistical significance in the comparison between “residential green” and “therapeutic garden” settings. Torku et al. [46] found no significant results in beta-band activity over all measurements.

Most studies investigating beta waves interpret the results to suggest that an increase in the beta frequency band is associated with a state of altered vigilance, attention or cognitive demand [14,37,40,43,44]. Reeves et al. [44] and Hassan et al. [40] propose that the increased power within the beta band during exposure to natural, as opposed to urban environments indicates improved attention. Baumann & Brooks-Cederqvist [14] indicate an increased beta frequency in more urbanized environments, suggesting a greater cognitive demand due to increased information processing. Similarly, Neale et al. [37] note the need for increased vigilance and visual attention during navigation in urban environments and attribute the observed increase in beta waves to this phenomenon. Olszewska-Guizzo et al. [43], in their exploration of TBA, characterize TBA as an indicator of attentional restoration. They claim that a low TBA indicates greater mental fatigue, thus explaining that the clinical group showed increased mental fatigue in the urban environment compared to the healthy control group. Two studies do not go into further detail on the interpretation of the beta band [39,46].

##### 3.4.1.3. Theta

Moreover, five studies investigated variations within the theta band [14,39,41,44,46]. Of the five studies examining theta power, four did not provide any information on the specific topographical localization of the effects [14,39,44,46]. In the evaluation of the theta band, two studies observed that more urbanized regions were associated with a decrease in the theta band [14,39], whereas one study indicated the contrary [41].

In Chen et al. [39], the decrease in overall theta power was shown in the difference between a traffic island and a forested campus, while in the study by Baumann & Brooks-Cederqvist [14] this effect was evident in a medium-density urban environment compared to a low-density urban environment. In contrast to this, Hopman et al. [41] found an inverse correlation, meaning that higher posterior and midfrontal theta activity was associated with more urbanized regions. The study [41] observed increased posterior and midfrontal theta power during exposure between campus buildings compared to measurements during a nature walk, particularly pronounced during eyes-closed conditions, although not significant during eyes-open conditions. However, two other studies found no significant differences in theta power between urban and natural environments [44,46].

In their assessment of the theta band, Reeves et al. [44] suggest that an increase in power across the theta-frequency might be associated with a state of relaxation and meditation. Similarly, Baumann & Brooks-Cederqvist [14] propose that the predominant theta activity in low-density urban environments can be interpreted as indicative of an awake yet relaxed state, suggesting lower physiological arousal compared to medium-density urban environments. No additional interpretations were provided by three other papers which included theta band data [39,41,46].

##### 3.4.1.4. Gamma and delta

Reeves et al. [44] did not find any significant differences in delta waves between urban and natural environments. Meanwhile, Chen et al. [39] found a decrease in delta waves during urban exposure compared to nature exposure, as indicated by the overall power correlation. Regarding gamma waves, this study did not report any specific results. Torku et al. [46] reported no significant results for gamma and delta band.

Reeves et al. [44] claimed that altered delta waves are associated with a state of altered consciousness. However, as no differences were identified between the urban and control environments, no further interpretation is provided. Torku et al. [46] and Chen et al. [39] offer no interpretation of the results regarding gamma or delta.

#### 3.4.2. Affective computing.

Among the 15 studies analyzed, EEG results were presented in affective categories by five studies [17,36,38,40,47]. These categories were determined by software using proprietary algorithms designed to filter and translate combinations of EEG signals into variables representing emotional states. While three studies used Emotiv’s “Affective Suite” [49] as software [17,36,38], another two studies [40,47] used an algorithm provided by Neurosky [50]. The “Affective Suite” software uses the variables “meditation”, “engagement”, “excitement” and “frustration”. The Neurosky software uses the categories “meditation/relaxation” and “attention”. Due to the use of proprietary algorithms, no information on the topographic localization of the effects is recorded in these studies.

When examining the first component, meditation, Aspinall et al. [38] reported higher levels of meditation in urban green spaces compared to urban shopping streets. This finding is consistent with that of Al-Barrak et al. [47], who observed increased levels of meditation in green control environments compared to supermarkets. Similarly, another study found increased meditation levels in bamboo forests compared to urban areas [40]. Two further studies [17,36] also attempted to define levels of meditation with the ‘Affective Suite’ but were unable due to poor data quality.

Investigations of “engagement” in the study of Aspinall et al. [38] showed a reduction in engagement in urban green compared to shopping street. In addition, Tilley et al. [36] detected a decrease in urban green compared to urban busy environments. Similarly, Neale et al. [17] reported higher levels of engagement in urban busy environments compared to urban green spaces, while also observing higher levels of engagement in urban green spaces compared to urban quiet environments. The order of exposure was irrelevant for the direction of the effect.

For the affective category “excitement”, Aspinall et al. [38] reported that walking through “urban green” and “urban busy” showed increased levels of excitement, while walking through more commercial districts showed decreased levels of excitement compared to the other districts. By contrast, the study of Neale et al. [17], observed a decrease in “urban busy” compared to urban green environment and an increase in “urban busy” compared to “urban quiet”. At the same time, however, no changes in levels of excitement could be identified between “urban green” and “urban quiet”. In terms of frustration levels, Aspinall et al. [38] reported higher levels in shopping streets compared to green spaces and commercial areas and a decrease in urban green spaces compared to shopping streets. Another study identified an increase in frustration in “urban green” compared to “urban busy” [36].

While Neale et al. [17] found no effects between “urban busy” and “urban green” or “urban busy” and “urban quiet” in the level of frustration, an increase in urban green was observed for the route between “urban green” and “urban quiet”.

For the last parameter “attention”, Al Barrak et al. [47] identified increased levels in supermarket environments in contrast to café or garden. In contrast, Hassan et al. [40] stated higher levels of attention in the bamboo forest as opposed to the city area.

#### 3.4.3. Brain functional connectivity.

Chen et al. [39] assessed functional connectivity across different exposures. A higher global EEG correlation was observed during nature exposure compared to urban exposure. Furthermore, similar results were found in the frequency domain functional topography in the theta band for both nature and urban exposure conditions. Finally, stronger functional connectivity networks were identified in the right hemisphere during nature exposure compared to urban exposure, with this effect being most pronounced in the theta band. The lateralization effect was stronger for the nature condition than for the urban condition in most brain regions, except for the P8/P7 and O2/O1 regions.

The researchers suggest that the functional connectivity findings could mean that the human brain is functionally connected when exposed to nature, allowing for specialized information processing and coordination between these areas. The small-world features of the functional network during nature experiences were particularly evident in the theta band, which is associated with the processing of new information, suggesting the possibility of a more efficient network for information processing in natural environments.

#### 3.4.4. Event related potentials.

In the study by Scanlon et al. [45], the test subjects had to solve an auditory oddball task on a bicycle and cycle through different environments, the event-related potentials were analyzed at the components N1, P2, and P3. The N1 component showed an increase in amplitude during the road condition compared to the park condition during the oddball task. This specific component showed a frontocentral distribution. Conversely, no significant differences in amplitude were observed at the P2 and P3 components during the oddball task between the roadway and other conditions. The P3 component was observed in the posterior (Pz) topographical location.

The authors suggest that the N1-P2 complex is associated with cognitive processes such as memory performance, working memory and semantic processing in context-dependent tasks. They propose that it reflects the suppression of the perception of irrelevant stimuli to enable discrimination of stimuli within the main task. Therefore, the lack of variation in P2 suggests that the increased noise from the road did not create a significant need to filter out background noise, or that the background noise remained the same in the urban or natural condition.

### 3.5. Further measures

While the review’s inclusion criteria only required EEG measurements, additional mental health parameters were also assessed. For example, four studies administered versions of the “Profile of Mood States” alongside EEG recordings, measuring the participants’ emotional states across multiple dimensions, e.g., tension, depression, anger, fatigue, confusion, and energy [39,42,43,48]. Furthermore, two studies used in-depth interviews to evaluate mental states asking about life stress or levels of anxiety and depression [36,44]. Objective measures, including cardiovascular and electrodermal activity metrics, were also collected in five instances [14,40,44,46,48]. Additionally, various tests examining emotional states across different domains were employed, such as the “Positive and Negative Affect Schedule” [44], the “State-Trait Anxiety Inventory” [40], or the “Perceived Restorativeness Scale” [39]. Moreover, this review scrutinized the inclusion of gender/sex aspects within the experimental studies. Only one paper incorporated gender into its analyses [41]. In the study, post hoc exploratory analyses included gender as a predictor in all models. While gender was acknowledged as a potential modifying variable in one study’s discussion, it was not further included in the analysis [46].

### 3.6. Quality assessment

The assessment of study quality was conducted using the EPHPP tool [35] for quantitative studies, chosen for its suitability for analyzing quantitative research within a public health framework. This tool evaluates various aspects including selection bias, study design, confounding variables, blinding, data collection methods, withdrawals and dropouts, intervention integrity, and analyses. Each component is assessed using specific questions (Q1-Q4) and can range between the levels: (1) strong (***), (2) moderate (**), and (3) weak (*). Subsequently, a global score is calculated, categorizing the study bias as weak, moderate, or strong. After the evaluation, all 15 studies received a “moderate” rating (Table 4). Details can be found in Supplementary material (S5 Table).

**Table 4:** Quality assessment according to the Effective Public Health Practice Project Tool.

Author, Year	Selection Bias	Study Design	Con-founders	Blin-ding	Data Collection	Withdrawals & Dropouts	Global Rating
Al-Barrak et al., 2017 [47]	*	**	**	**	**	**	**
Aspinall et al., 2015 [38]	*	**	*	*	***	**	**
Baumann & Brooks-Cederqvist, 2023 [14]	***	**	*	**	***	***	**
Chen et al., 2016 [39]	**	**	**	**	***	**	**
Elsadek et al., 2019 [48]	*	**	*	**	**	**	**
Hassan et al., 2018 [40]	**	**	*	*	***	**	**
Hopman et al., 2020 [41]	*	**	*	*	**	***	**
Neale et al., 2017 [17]	**	**	**	*	**	**	**
Neale et al., 2020 [37]	*	**	*	**	**	**	**
Olszewska-Guizzo et al., 2020 [42]	*	**	*	**	**	***	**
Olszewska-Guizzo et al., 2022 [43]	*	**	*	**	**	***	**
Reeves et al., 2019 [44]	*	**	**	***	**	***	**
Scanlon et al., 2020 [45]	*	**	*	**	**	***	**
Tilley et al., 2017 [36]	**	**	*	*	**	***	**
Torku et al., 2022 [46]	*	**	*	*	**	***	**

* weak; ** moderate; *** strong

## 4. Discussion

The aim of this systematic review was to synthesize reported differences in brain electrical signals due to exposure to different (urban) environments using mobile EEG published between 2013 and 2023. This discussion aims to evaluate the findings within the existing body of research and beyond. We found 15 studies fulfilling inclusion criteria and observed large heterogeneity regarding experimental paradigms, study participants, urban exposures and results

### 4.1. General discussion of results

In summary, the current review examined 15 interventional studies employed in a within-subject or between-subject design, predominantly involving elderly individuals and university students as participants. These comparisons primarily explore brain electrical changes in urban environments, such as busy intersections or residential areas, and greener control exposures like therapeutic gardens, bamboo forests, or green facades. The alpha, beta, and theta frequency bands were the most frequently analyzed, with less frequent analyses conducted on the gamma and delta bands. However, the investigated bands exhibit a large inconsistency in results regarding directionality of effects and their interpretation. While certain studies indicate an increase in spectral power in urban settings compared to less urbanized regions, others demonstrate a decrease. Distinct emotional states were associated with various frequency bands in the discussed studies. Alpha activity was commonly interpreted as indicative of relaxation or improved attention, whereas beta activity was described as signaling heightened vigilance, attention, cognitive demand, or mental fatigue. The theta band was typically associated with a state of wakeful relaxation. Due to the variability in findings, assigning specific emotional states to individual exposures based on the selected studies is challenging.

Several factors may contribute to the heterogeneous results observed across studies. First, it is important to acknowledge the diverse geographical and cultural contexts in which the studies were conducted. Urban areas around the world are shaped by specific cultural and contextual characteristics that can significantly influence the nature of exposure to various environmental factors. For example, Southeast Asian cities differ in terms of population density, building density or land use mix - the combination of residential, industrial, commercial, open space, community facilities, parks and schools [51]. The specific characteristics, and therefore unique patterns of potential stressors and urban resources, may limit direct comparability. Hence, generic descriptions such as “busy intersection” may indicate a different environment depending on the geographical context or personal experiences, emphasizing the need for accurate exposure descriptions. To enable a meaningful analysis and discussion of results within cultural or geographical contexts, it is essential that geographical data, such as country and city, are consistently reported - a criterion that was not met in some of the studies we identified. Standardized reporting of characteristics, such as building and population density, would enhance comparability across studies. Tonne et al. [52] also describe urbanity as a latent variable that cannot be directly measured, and they highlight that global heterogeneity and the consequent lack of established comparative units pose a challenge for the science of urban health research.

While some studies utilize various tools to precisely classify and operationalize urban exposure, it is striking that the majority of experimental studies lack detailed descriptions of exposure. This is a common issue not only in research examining the effects of urban environments on brain electrical signals but also in studies investigating the influence of green spaces on mental health [53]. In a review investigating the current methods used for researching green spaces on mental health, Freymueller et al. [53] suggest that the use of reporting guidelines for the description of green exposures could serve as a valuable tool to increase research quality and improve reproducibility in environmental mental health studies. For future in-situ EEG studies, reporting guidelines, for example when urban parks are involved as a compared exposure, could also be discussed to increase traceability of exposures. In addition to accurately characterizing environments, it is crucial to consider the technical aspects that can facilitate the operationalization of exposure. For instance, integrating additional sensors and data sources to measure light levels, ambient noise levels [47], or other environmental factors could serve as valuable tools and offer insights into addressing the existing heterogeneity in results [52].

Another aspect that might affect the reliability of mobile EEG studies is the challenge to obtain high-quality EEG data when conducting measurements in real-world settings. The measurement of brain activity is inherently affected by noise and artifacts, which occur during movement and muscle activity, through sweat bridges, electrodes or cables movements, cardiac activity, and eye movement [54]. A major challenge is optimizing the signal-to-noise ratio, given that mobile participation tends to introduce additional noise [55]. Thus, consumer-grade devices, as shown in most of the studies included in the review, usually are prone to poor data quality in real-life settings [56]. Despite the inherent challenges associated with mobile EEG, advances in this technology, such as newly developed algorithms, are becoming increasingly prominent. For example, the brMEGA algorithm [57] supports the automated detection and reduction of cardiogenic artifacts by using non-linear time-frequency analysis and machine learning techniques, helping to enhance signal quality in mobile EEG applications.

In a recent critical review by Gramann [31], the weak methodological approaches and implementation in many neurourbanistic mobile EEG studies were extensively debated, and similar issues are evident in the studies we identified. A key methodological concern in recent studies is the prevalent use of proprietary algorithms for data processing. Specifically, five studies rely on this approach, where the processing algorithms and specifications essential to feature extraction are not accessible for critical evaluation, raising concerns about replicability. Consequently, results from these black-box methods lacking transparency in algorithmic details, along with missing statistics on cleaned or interpolated data, hinder robust analysis. Approximately one-third of the studies we identified, including all those categorized within the “affective computing chapter”, utilized this method.

Gramann [31] also highlighted the significant methodological differences between low-density and high-density EEG systems. He criticizes that 82.2% of the studies in his review utilized fewer than 20 electrodes. Similarly, our analysis revealed that all studies employed fewer than 20 electrodes, with two studies using only a single electrode. This limitation aligns with the inability to analytically exclude non-neural physiological artifacts, such as eye movements or facial muscle contractions, as well as motion-related artifacts in ambulatory settings. These challenges persist even in systems using up to 14 electrodes, such as the Emotiv Epoc, a consumer-grade EEG device frequently employed in the studies we analyzed. This makes it difficult to apply advanced processing tools like Independent Component Analysis (ICA) effectively. When employed correctly, techniques like ICA are able to separate the effects of eye movements, muscle activity, or electrical artifacts from the brain activity under investigation, thereby ensuring a more accurate analysis, according to Klug and Gramann [58]. Regardless of the increased risk of artifact occurrence in mobile experiments, ICA remains a valuable tool for artifact removal. The authors observed variations in decomposition efficiency under varying hardware (e.g., high-pass filter settings, number of electrodes) and experimental conditions like moving exposure, leading them to formulate guidelines. These guidelines can serve as crucial reference points for mobile studies and contribute to enhancing data quality [58].

The alternative, i.e., exposure to virtual realities in shielded laboratories, as has often been used in the past to investigate urban settings, comes with major limitations [23,59]. A laboratory setting might be too rigid and artificial to properly reproduce real emotional experiences of everyday life [60]. Only certain sensory impressions can be simulated in the laboratory setting, but a holistic environmental experience can only be guaranteed through multisensory immersion [39]. Future research should consider this tradeoff between external validity through the ecological setting with limited data quality and virtual laboratory studies with higher internal validity with better data quality replicability when investigating the real connections between mental states and urban settings.

In addition, there are some methodological and conceptual aspects that have not yet been sufficiently covered in the current study situation and thus expose research gaps. The analysis of the different populations included in the review reveals that adolescents are not explicitly included in the 15 studies. However, previous neurourbanistic research indicates that adolescence may represent a crucial period for neurodevelopmental changes and increased sensitivity to urban stressors [61,62]. Lederbogen and colleagues [62] suggested that adolescence may be a critical period for the formation of neural circuits involved in (urban) stress regulation and that brain regions such as the perigenual anterior cingulate cortex may have a developmental vulnerability. As such, adolescents may be a particularly relevant group for in-situ EEG experiments, suggesting that future research may prioritize investigations involving this demographic. This may even help to understand the underlying mechanisms linking urban upbringing to mental health outcomes.

Another demographic aspect that is rarely considered when analyzing the studies is the gender-specific differences. However, there is evidence that gender may influence the processing of emotional stimuli, suggesting potentially relevant variations in how individuals of different genders respond to such stimuli. For example, it has been shown that women share more similar EEG patterns among them when emotions are evoked, while men have more individual variances among their EEG patterns [63]. To accurately assess the influence of the urban environment, it is essential to incorporate these variations into the study design, enabling a nuanced, quantitative representation of this variable, beyond binary categorization, and promoting consistent reporting standards.

The predominant approach in many of the included studies is to quantify urbanity by assessing the environmental conditions and exposures based on the degree of greenery or built (grey) environments in certain areas. As a result, descriptions of exposures often focus on physical environmental factors, such as comparing moderate-density to low-density areas or contrasting vegetated building walls with concrete building walls. However, numerous other aspects of urbanity, like social aspects may significantly influence stress development and therefore mental health. For instance, advancements in mobile EEG technology offer novel opportunities to analyze such social dynamics within real world contexts [25]. This is particularly relevant given that Adli & Schöndorf [4] hypothesize that social stress, particularly stress resulting from high social density combined with experiences of social isolation, could have a significant impact on urban mental health. Jalilisadrabad et al. [64] analyzed research investigating the impact of several categories of urban stressors regarding mental health. The authors found that urban stress was mostly induced by social stressors. While environmental stressors were the second most important group of stressors, economic, housing or cultural stressors were also relevant for the impact of the urban environment on mental health. In order to assess the multitude of different stressor source for mental health in the urban environment, it is necessary to implement appropriate assessment tools or technical solutions. Despite the indications that stress arising from the relationship between the individual and the social environment plays a special role, it is still unclear under which conditions urban stressors lead to pathogenic stress and when they contribute to the social development of the individual [4]. Therefore, issues like urban crowding effects may emerge as crucial topics for research, which was already conducted in a VR-setting [65] and could also be used to further investigate how the mechanisms of density stress and isolation stress might be related to mental health and wellbeing. Yet, these stressors are not comprehensively addressed in current research, highlighting the incomplete mapping of urbanity. Future studies should endeavor to include these aspects more thoroughly.

In summary, this area of research is still in its early stages, with ongoing developments needed both in technological capabilities and the depth of content exploration.

### 4.2. Strengths and limitations

One of the strengths of this systematic literature review is its comprehensive search strategy in the four databases PubMed/MEDLINE, Embase, PsycInfo, and CINAHL, which included a wide range of search terms. Additionally, the screening, extraction, and quality assessment processes were consistently validated and independently executed by two authors, enhancing the review’s reliability. Moreover, the study was preregistered with PROSPERO, enhancing the transparency and traceability of the methodological framework employed. However, the inclusion criteria limited the scope to English and German studies, which, although covering a substantial proportion of the literature, may have resulted in the omission of individual studies conducted in other languages. Multilingual teams could help to create a more global and broader perspective. A further limitation of the review is evident in the implementation of the quality assessment. Although the EPHPP assessment is an instrument that has already been used for other reviews of neurourbanistic or mobile research methods [24]. It is noticeable when analyzing the studies presented that almost all studies were rated “moderate”. However, the standardized questions of the quality assessments are not optimally adapted to this type of study, meaning that certain criteria of the evaluation instrument can never be fully met. The EPHPP tool may not be ideal for evaluating mobile EEG studies in urban environments due to its emphasis on randomization and large sample sizes (see Selection Bias Q1). In real-world urban settings, achieving full randomization and large samples is challenging due to technical and logistical constraints. This may unfairly lower ratings for studies with appropriate methods for this context. Additionally, EPHPP does not account for environmental factors that can affect stress measurements. Adding items to assess environmental context and technical challenges could improve its applicability to this research. For future approaches, it would be valuable to explore whether combining assessment tools could improve sensitivity in identifying differences within ecological intervention comparisons and provide a more precise evaluation of study quality.

## 5. Conclusion

This review provides a comprehensive insight into the current state of mobile EEG studies in urban mental health research. Technical limitations in deploying mobile EEGs in real-world settings pose challenges for precise measurement. Moreover, certain demographic groups, such as adolescents, remain understudied, while aspects like gender differences lack sufficient investigation. Additionally, considerable heterogeneity within the results exists among the studies, possibly due to inadequate description of exposures and limited comparability of the employed experimental designs. Despite these limitations, in-situ EEG holds promise for offering valuable insights in the future. Especially if future studies emphasize methodological rigor, address demographic gaps, and enhance data collection quality in real-world settings, they could aid in the comprehension of emotional and cognitive states, subsequently contributing to urban mental health research.

## Supporting information

S1 Text preregistered Protocol PROSPERO. (PDF)

S1 Table PRISMA Checklist. (DOCX)

S2 Table Search strategy per database. (DOCX)

S3 Table Individual Screening decisions. (XLSX)

S4 Table Extraction table. (XLSX)

S5 Table Quality Assessment. (XLSX)
