# Supplementary material for: Exploring urban mental health using mobile EEG – a systematic review
Source: PLOS Ment Health. 2025 Apr 4;2(4):e0000203. doi: 10.1371/journal.pmen.0000203 (PMC12798177; doi:10.1371/journal.pmen.0000203)
Supplement: S1 Text — (PDF) [file pmen.0000203.s001.pdf]

Systematic review

A list of fields that can be edited in an update can be found here

1. * Review title.

Give the title of the review in English

Exploring urban mental health using mobile EEG – a systematic review

2. Original language title.

For reviews in languages other than English, give the title in the original language. This will be displayed with the English language title.

3. * Anticipated or actual start date.

Give the date the systematic review started or is expected to start.

10/09/2023

4. * Anticipated completion date.

Give the date by which the review is expected to be completed.

15/02/2024

5. * Stage of review at time of this submission.

This field uses answers to initial screening questions. It cannot be edited until after registration.

Tick the boxes to show which review tasks have been started and which have been completed.

Update this field each time any amendments are made to a published record.

The review has not yet started: No

Review stage	Started	Completed
Preliminary searches	Yes	No
Piloting of the study selection process	Yes	No
Formal screening of search results against eligibility criteria	No	No
Data extraction	No	No
Risk of bias (quality) assessment	No	No
Data analysis	No	No

Provide any other relevant information about the stage of the review here.

6. * Named contact.

The named contact is the guarantor for the accuracy of the information in the register record. This may be any member of the review team.

Ben Senkler

Email salutation (e.g. "Dr Smith" or "Joanne") for correspondence:

Mr Senkler

7. * Named contact email.

Give the electronic email address of the named contact.

ben.senkler@gmail.com

8. Named contact address

Give the full institutional/organisational postal address for the named contact.

Boettcherstrasse 41, 48165 Muenster, Germany

9. Named contact phone number.

Give the telephone number for the named contact, including international dialling code.

+4915751769978

10. * Organisational affiliation of the review.

Full title of the organisational affiliations for this review and website address if available. This field may be

completed as 'None' if the review is not affiliated to any organisation.

Bielefeld University - Medical Faculty OWL - AG 1 Sustainable Environmental Health Sciences

Organisation web address:

https://www.uni-bielefeld.de/fakultaeten/medizin/fakultaet/arbeitsgruppen/environment/

11. * Review team members and their organisational affiliations.

Give the personal details and the organisational affiliations of each member of the review team. Affiliation refers to groups or organisations to which review team members belong. **NOTE: email and country now MUST be entered for each person, unless you are amending a published record. PLEASE USE AN INSTITUTIONAL EMAIL ADDRESS IF POSSIBLE.**

Mr Ben Senkler. Bielefeld University - AG 1 Sustainable Environmental Health Sciences
Mr Toivo Glatz. Charite - Universitätsmedizin Berlin - Institute of Public Health
Mr Timothy McCall. Bielefeld University - AG 1 Sustainable Environmental Health Sciences

12. * Funding sources/sponsors.

Details of the individuals, organizations, groups, companies or other legal entities who have funded or sponsored the review.

Not applicable

Grant number(s)

State the funder, grant or award number and the date of award

Not applicable

13. * Conflicts of interest.

List actual or perceived conflicts of interest (financial or academic).

None

14. Collaborators.

Give the name and affiliation of any individuals or organisations who are working on the review but who are not listed as review team members. **NOTE: email and country must be completed for each person, unless you are amending a published record.**

15. * Review question.

State the review question(s) clearly and precisely. It may be appropriate to break very broad questions down into a series of related more specific questions. Questions may be framed or refined using PI(E)COS or similar where relevant.

What are the results of mobile EEG studies on the relationship between urban living and mental health over the past ten years?

16. * Searches.

State the sources that will be searched (e.g. Medline). Give the search dates, and any restrictions (e.g.

language or publication date). Do NOT enter the full search strategy (it may be provided as a link or attachment below.)

For a comprehensive range of recent literature, medical databases as well as specific psychological databases will be used. Consequently, CINAHL, PubMed/MEDLINE, PsycINFO, and Embase will be consulted and systematically searched according to a predefined search string. The searchdate is the 27.09.2023.

Only publications from the years 2013-2023 will be considered. In terms of language restrictions only German or English studies will be included.

Due to the short processing time, no re-run before the final analyses is planned.

Unpublished studies will not be sought.

17. URL to search strategy.

Upload a file with your search strategy, or an example of a search strategy for a specific database, (including the keywords) in pdf or word format. In doing so you are consenting to the file being made publicly accessible. Or provide a URL or link to the strategy. Do NOT provide links to your search **results**.

Alternatively, upload your search strategy to CRD in pdf format. Please note that by doing so you are consenting to the file being made publicly accessible.

Do not make this file publicly available until the review is complete

18. * Condition or domain being studied.

Give a short description of the disease, condition or healthcare domain being studied in your systematic review.

Stress-related mental illnesses, which are particularly common in cities, and changes in cognitive abilities are examined. However, the review also focuses on emotional states that do not represent a manifest illness, but are closely related to it. Consequently, in addition to psychoses, depression and anxiety disorders and cognitive performance, affective states, mood, fatigue or states of tension caused by stress are primarily examined.

19. * Participants/population.

Specify the participants or populations being studied in the review. The preferred format includes details of both inclusion and exclusion criteria.

Included: urban populations or populations spending time in urban regions. No restrictions in age or health status.

Excluded: animals

20. * Intervention(s), exposure(s).

Give full and clear descriptions or definitions of the interventions or the exposures to be reviewed. The preferred format includes details of both inclusion and exclusion criteria.

The exposures studied relate to the environmental influences of urban regions.

1) Influences of the built environment are examined. This includes exposures like looking at skyscrapers, walking along busy/noisy streets etc..

2) Contact to urban natural environments. This includes urban blue and green spaces, parks, urban forests but also exposure to vertical green facades.

3) Social influences of urban life such as crowding or residential density as well as the transgression of personal space are also examined.

All types of virtual urban environments (VR/Slideshows/Videos) are excluded as exposure.

21. * Comparator(s)/control.

Where relevant, give details of the alternatives against which the intervention/exposure will be compared (e.g. another intervention or a non-exposed control group). The preferred format includes details of both inclusion and exclusion criteria.

A comparison group is not necessary for the scope of this study.

22. * Types of study to be included.

Give details of the study designs (e.g. RCT) that are eligible for inclusion in the review. The preferred format includes both inclusion and exclusion criteria. If there are no restrictions on the types of study, this should be stated.

Included: experimental/interventional studies, (observational studies)

Excluded: reviews and meta-analyses.

23. Context.

Give summary details of the setting or other relevant characteristics, which help define the inclusion or exclusion criteria.

Only studies conducted in an urban setting are included. Consequently, at least one urban feature, such as a busy street, tall buildings, or similar environmental features, must be examined as the primary exposure.

24. * Main outcome(s).

Give the pre-specified main (most important) outcomes of the review, including details of how the outcome is defined and measured and when these measurement are made, if these are part of the review inclusion criteria.

The main outcome are the brain electrical signals measured by electroencephalography and their derivations regarding mental states.

Measures of effect

Please specify the effect measure(s) for you main outcome(s) e.g. relative risks, odds ratios, risk difference, and/or 'number needed to treat.

All measurements using in-situ/mobile EEG are examined. Depending on the scope of the research question, various effect measures such as event-related potentials, power spectral density or connectivity measures etc. can be examined.

25. * Additional outcome(s).

List the pre-specified additional outcomes of the review, with a similar level of detail to that required for main outcomes. Where there are no additional outcomes please state 'None' or 'Not applicable' as appropriate to the review

Not applicable

Measures of effect

Please specify the effect measure(s) for you additional outcome(s) e.g. relative risks, odds ratios, risk difference, and/or 'number needed to treat.

Not applicable

26. * Data extraction (selection and coding).

Describe how studies will be selected for inclusion. State what data will be extracted or obtained. State how this will be done and recorded.

Study selection

After aggregating all search terms into a unique search strategy, the records from the databases are initially extracted into Rayyan, an AI-powered systematic review tool. At least two researchers screen all records separately for inclusion. After the finished screening process the selected records will be compared. Any disagreements about inclusion or exclusion will be finally decided by a third researcher.

Data extraction

The data extraction will be designed according to the number of included studies after full-text screening. With a higher number (n=50) of included studies, the interrater reliability will be determined with 10% of the studies in order to divide the data extraction between the reviewers. If the number of studies is n50, the researchers will extract all studies independently. In case of discrepancies, another researcher will be consulted.

- Extracted data: sample sizes and study design, (mean) age, setting (country), exposure, main results. Further elements will be added after the completion of the screening-process.

- Missing data will be recorded and reported.

- The data is collected in an Excel spreadsheet.

27. * Risk of bias (quality) assessment.

State which characteristics of the studies will be assessed and/or any formal risk of bias/quality assessment tools that will be used.

The quality assessment will be conducted using the "EPHPP Quality Assessment tool". Hence, selection bias, study design, confounding, data collection methods, withdrawl and dropouts and the data analysis will be examined. The quality assessment is carried out by at least two researchers. Disagreements regarding classification will be resolved by an independent researcher. The quality indicators will be reported but will not be considered in the data synthesis.

28. * Strategy for data synthesis.

Describe the methods you plan to use to synthesise data. This **must not be generic text** but should be **specific to your review** and describe how the proposed approach will be applied to your data. If meta-analysis is planned, describe the models to be used, methods to explore statistical heterogeneity, and software package to be used.

Data is reported narratively using the extraction table. No effect measures are synthesized directly; rather, the results of the individual studies are reported in summary.

29. * Analysis of subgroups or subsets.

State any planned investigation of ‘subgroups’. Be clear and specific about which type of study or participant will be included in each group or covariate investigated. State the planned analytic approach.

No subgroup analyzes are initially planned.

30. * Type and method of review.

Select the type of review, review method and health area from the lists below.

Type of review

Cost effectiveness

No

Diagnostic

No

Epidemiologic

No

Individual patient data (IPD) meta-analysis

No

Intervention

No

Living systematic review

No

Meta-analysis

No

Methodology

No

Narrative synthesis

Yes

Network meta-analysis

No

Pre-clinical

No

Prevention

No

Prognostic

No

Prospective meta-analysis (PMA)

No

Review of reviews

No

Service delivery

No

Synthesis of qualitative studies

No

Systematic review

Yes

Other

No

Health area of the review

Alcohol/substance misuse/abuse

No

Blood and immune system

No

Cancer

No

Cardiovascular

No

Care of the elderly

No

Child health

No

Complementary therapies

No

COVID-19

No

Crime and justice

No

Dental

No

Digestive system

No

Ear, nose and throat

No

Education

No

Endocrine and metabolic disorders

No

Eye disorders

No

General interest

No

Genetics

No

Health inequalities/health equity

No

Infections and infestations

No

International development

No

Mental health and behavioural conditions

Yes

Musculoskeletal

No

Neurological

No

Nursing

No

Obstetrics and gynaecology

No

Oral health

No

Palliative care

No

Perioperative care

No

Physiotherapy

No

Pregnancy and childbirth

No

Public health (including social determinants of health)

No

Rehabilitation

No

Respiratory disorders

No

Service delivery

No

Skin disorders

No

Social care

No

Surgery

No

Tropical Medicine

No

Urological

No

Wounds, injuries and accidents

No

Violence and abuse

No

31. Language.

Select each language individually to add it to the list below, use the bin icon to remove any added in error.
English

There is not an English language summary

32. * Country.

Select the country in which the review is being carried out. For multi-national collaborations select all the countries involved.

Germany

33. Other registration details.

Name any other organisation where the systematic review title or protocol is registered (e.g. Campbell, or The Joanna Briggs Institute) together with any unique identification number assigned by them. If extracted data will be stored and made available through a repository such as the Systematic Review Data Repository (SRDR), details and a link should be included here. If none, leave blank.

34. Reference and/or URL for published protocol.

If the protocol for this review is published provide details (authors, title and journal details, preferably in Vancouver format)

Add web link to the published protocol.

Or, upload your published protocol here in pdf format. Note that the upload will be publicly accessible.

No I do not make this file publicly available until the review is complete

Please note that the information required in the PROSPERO registration form must be completed in full even if access to a protocol is given.

35. Dissemination plans.

Do you intend to publish the review on completion?

Yes

Give brief details of plans for communicating review findings.?

The results will be published as open access, making them available to the general public.

36. Keywords.

Give words or phrases that best describe the review. Separate keywords with a semicolon or new line. Keywords help PROSPERO users find your review (keywords do not appear in the public record but are included in searches). Be as specific and precise as possible. Avoid acronyms and abbreviations unless these are in wide use.

Urban mental health; mobile EEG; urban environmental psychology; neurourbanism

37. Details of any existing review of the same topic by the same authors.

If you are registering an update of an existing review give details of the earlier versions and include a full bibliographic reference, if available.

38. * Current review status.

Update review status when the review is completed and when it is published.New registrations must be ongoing so this field is not editable for initial submission.

Please provide anticipated publication date

Review_Ongoing

39. Any additional information.

Provide any other information relevant to the registration of this review.

The anticipated publication date is 15.05.2024.

40. Details of final report/publication(s) or preprints if available.

Leave empty until publication details are available OR you have a link to a preprint (NOTE: this field is not editable for initial submission). List authors, title and journal details preferably in Vancouver format.

Give the link to the published review or preprint.
