# Supplementary material for: Exploring urban mental health using mobile EEG – a systematic review
Source: PLOS Ment Health. 2025 Apr 4;2(4):e0000203. doi: 10.1371/journal.pmen.0000203 (PMC12798177; doi:10.1371/journal.pmen.0000203)
Supplement: S2 Table — (DOCX) [file pmen.0000203.s003.docx]

**Supplementary Material Table S1
search strategy Pubmed; Embase; PsycINFO; CINAHL**

| **Database** | **Number of results** | **Searchstring** |
| --- | --- | --- |
| **Medline (Pubmed)** | 1242  (27.09.2023) | (("urban*"[Title/Abstract] OR "city*"[Title/Abstract] OR "citie*"[Title/Abstract] OR "Cities"[MeSH Terms] OR "metropolitan*"[Title/Abstract] OR "residential*"[Title/Abstract] OR "agglomeration*"[Title/Abstract] OR "town*"[Title/Abstract] OR "built environment*"[Title/Abstract] OR "built environment"[MeSH Terms] OR "alley*"[Title/Abstract] OR "facade*"[Title/Abstract] OR "intersection*"[Title/Abstract] OR "plaza*"[Title/Abstract] OR "road*"[Title/Abstract] OR "sidewalk*"[Title/Abstract] OR "street*"[Title/Abstract] OR "high building*"[Title/Abstract] OR "skyscraper*"[Title/Abstract] OR "traffic noise*"[Title/Abstract] OR "noise, transportation"[MeSH Terms] OR "railway noise*"[Title/Abstract] OR "highway*"[Title/Abstract] OR "blue space*"[Title/Abstract] OR "forest*"[Title/Abstract] OR "forests"[MeSH Terms] OR "greenspace*"[Title/Abstract] OR "green space*"[Title/Abstract] OR "park*"[Title/Abstract] OR "parks, recreational"[MeSH Terms] OR "river*"[Title/Abstract] OR "rivers"[MeSH Terms] OR "tree*"[Title/Abstract] OR "trees"[MeSH Terms] OR "green infrastructure*"[Title/Abstract] OR "vegetation*"[Title/Abstract] OR "greenway*"[Title/Abstract] OR "bluespace*"[Title/Abstract] OR "vertical green*"[Title/Abstract] OR "population density*"[Title/Abstract] OR "population density"[MeSH Terms] OR "peripersonal space*"[Title/Abstract] OR "overcrowding*"[Title/Abstract] OR "crowding"[MeSH Terms] OR "residential density*"[Title/Abstract]) AND ("EEG"[Title/Abstract] OR "electroencephalograph*"[Title/Abstract] OR "electroencephalography"[MeSH Terms] OR "mobile electroencephalograph*"[Title/Abstract] OR "mobile eeg*"[Title/Abstract] OR "meeg*"[Title/Abstract]) AND ("mental health*"[Title/Abstract] OR "mental health"[MeSH Terms] OR "psychosis"[Title/Abstract] OR "psychotic disorders"[MeSH Terms] OR "anxiety*"[Title/Abstract] OR "anxiety"[MeSH Terms] OR "attention*"[Title/Abstract] OR "attention"[MeSH Terms] OR "restoration*"[Title/Abstract] OR "calm*"[Title/Abstract] OR "cognition*"[Title/Abstract] OR "cognition"[MeSH Terms] OR "cognitive function*"[Title/Abstract] OR "depression*"[Title/Abstract] OR ("depressive disorder"[MeSH Terms] OR "depression"[MeSH Terms]) OR "emotional regulation*"[Title/Abstract] OR "emotional regulation"[MeSH Terms] OR "fatigue*"[Title/Abstract] OR "fatigue"[MeSH Terms] OR "fear*"[Title/Abstract] OR "fear"[MeSH Terms] OR "internalizing disorder*"[Title/Abstract] OR "relax*"[Title/Abstract] OR "relaxation"[MeSH Terms] OR "rest*"[Title/Abstract] OR "rest"[MeSH Terms] OR "sleep*"[Title/Abstract] OR "sleep"[MeSH Terms] OR "stress*"[Title/Abstract] OR "stress, psychological"[MeSH Terms] OR "wellbeing*"[Title/Abstract] OR "well being*"[Title/Abstract] OR "well being*"[Title/Abstract] OR "worry*"[Title/Abstract] OR "mood*"[Title/Abstract] OR "emotional regulation"[MeSH Terms])) AND ((y_10[Filter]) AND (english[Filter] OR german[Filter])) |
| **Embase** | 2854  (27.09.2023) | (('urban*':ab,ti OR 'urban areas'/exp OR 'city*':ab,ti OR 'citie*':ab,ti OR 'City'/exp OR 'metropolitan*':ab,ti OR 'residential*':ab,ti OR 'agglomeration*':ab,ti OR 'town*':ab,ti OR 'built environment*':ab,ti OR 'built environment'/exp OR 'alley*':ab,ti OR 'facade*':ab,ti OR 'intersection*':ab,ti OR 'plaza*':ab,ti OR 'road*':ab,ti OR 'sidewalk*':ab,ti OR 'street*':ab,ti OR 'high building*':ab,ti OR 'skyscraper*':ab,ti OR 'traffic noise*':ab,ti OR 'traffic noise'/exp OR 'railway noise*':ab,ti OR 'highway*':ab,ti OR 'highway'/exp OR 'blue space*':ab,ti OR 'bluespace*':ab,ti OR 'forest*':ab,ti OR 'forest'/exp OR 'greenspace*':ab,ti OR 'green space*':ab,ti OR 'park*':ab,ti OR 'river*':ab,ti OR 'rivers'[MeSH Terms] OR 'tree*':ab,ti OR 'tree' OR 'green infrastructure*':ab,ti OR 'vegetation*':ab,ti OR 'greenway*':ab,ti OR 'vertical green*':ab,ti OR 'population density*':ab,ti OR 'population density'/exp OR 'peripersonal space*':ab,ti OR 'overcrowding*':ab,ti OR 'crowding (area)'/exp OR 'residential density*':ab,ti) AND ('EEG':ab,ti OR 'electroencephalograph*':ab,ti OR 'electroencephalography'/exp OR 'mobile electroencephalograph*':ab,ti OR 'meeg*':ab,ti) AND ('mental health*':ab,ti OR 'mental health'/exp OR 'psychosis':ab,ti OR 'psychosis'/exp OR 'anxiety*':ab,ti OR 'anxiety'/exp OR 'attention*':ab,ti OR 'attention'/exp OR 'restoration*':ab,ti OR 'calm*':ab,ti OR 'cognition*':ab,ti OR 'cognition'/exp OR 'cognitive function*':ab,ti OR 'depression*':ab,ti OR 'depression'/exp OR 'emotional regulation*':ab,ti OR 'emotional regulation'/exp OR 'fatigue*':ab,ti OR 'fatigue'/exp OR 'fear*':ab,ti OR 'fear'/exp OR 'internalizing disorder*':ab,ti OR 'relax*':ab,ti OR 'relaxation'/exp OR 'rest*':ab,ti OR 'sleep*':ab,ti OR 'sleep'/exp OR 'stress*':ab,ti OR 'mental stress'/exp OR 'wellbeing*':ab,ti OR 'well being*':ab,ti OR 'well-being*':ab,ti OR 'wellbeing'/exp 'worry*':ab,ti OR 'mood*':ab,ti OR 'mood'/exp AND *([english]/lim OR [german]/lim)* AND (2013:py OR 2014:py OR 2015:py OR 2016:py OR 2017:py OR 2018:py OR 2019:py OR 2020:py OR 2021:py OR 2022:py OR 2023:py) |
| **PsycINFO** | 393  (27.09.2023) | ((TI "urban*" OR AB "urban*" OR TI "city*" OR AB "city*" OR TI "citie*" OR AB "citie*" OR TI "Cities" OR AB "Cities" OR TI "metropolitan*" OR AB "metropolitan*" OR TI "residential*" OR AB "residential*" OR TI "agglomeration*" OR AB "agglomeration*" OR TI "town*" OR AB "town*" OR MA "Towns" OR TI "built environment*" OR AB "built environment*" OR MA "built environment" OR TI "alley*" OR AB "alley*" OR TI "facade*" OR AB "facade*" OR TI "intersection*" OR AB "intersection*" OR TI "plaza*" OR AB "plaza*" OR TI "road*" OR AB "road*" OR TI "sidewalk*" OR AB "sidewalk*" OR TI "street*" OR AB "street*" OR TI "high building*" OR AB "high building*" OR TI "skyscraper*" OR AB "skyscraper*" OR TI "traffic noise*" OR AB "traffic noise*" OR TI "railway noise*" OR AB "railway noise*" OR TI "highway*" OR AB "highway*" OR TI "forest*" OR AB "forest*" OR TI "greenspace*" OR AB "greenspace*" OR TI "green space*" OR AB "green space*" OR TI "park*" OR AB "park*" OR TI "river*" OR AB "river*" OR TI "tree*" OR AB "tree*" OR TI "trees" OR AB "trees" OR TI "green infrastructure*" OR AB "green infrastructure*" OR TI "vegetation*" OR AB "vegetation*" OR TI "greenway*" OR AB "greenway*" OR TI "bluespace*" OR AB "bluespace*" OR TI "blue space*" OR AB "blue space*" OR TI "vertical green*" OR AB "vertical green*" OR TI "population density*" OR AB "population density*" OR MA "Social Density" OR TI "peripersonal space*" OR AB "peripersonal space*" OR TI "overcrowding*" OR AB "overcrowding*" OR MA "crowding" OR TI "residential density*" OR AB "residential density*") AND (TI "EEG" OR AB "EEG" OR TI "electroencephalograph*" OR AB "electroencephalograph*" OR MA "electroencephalography" OR TI "meeg*" OR AB "meeg*") AND (TI "mental health*" OR AB "mental health*" OR MA "Mental health" OR TI "psychosis" OR AB "psychosis" OR MA "Psychosis" OR TI "anxiety*" OR AB "anxiety*" OR MA "Anxiety" OR TI "attention*" OR AB "attention*" OR MA "Attention" OR TI "restoration*" OR AB "restoration*" OR TI "calm*" OR AB "calm*" OR TI "cognition*" OR AB "cognition*" OR MA "Cognition" OR TI "cognitive function*" OR AB "cognitive function*" OR TI "Depression*" OR AB "Depression*" OR MA "Depression (Emotion)" OR TI "depressive disorder" OR AB "depressive disorder" OR TI "emotional regulation*" OR AB "emotional regulation*" OR MA "Emotional Regulation*" OR TI "fatigue*" OR AB "fatigue*" OR MA "fatigue" OR TI "fear*" OR AB "fear*" OR MA "Fear" OR TI "internalizing disorder*" OR AB "internalizing disorder*" OR TI "relax*" OR AB "relax*" OR MA "Relaxation" OR TI "rest*" OR AB "rest*" OR TI "sleep*" OR AB "sleep*" OR MA "Sleep" OR TI "stress*" OR AB "stress*" OR MA "Stress" OR TI "wellbeing*" OR AB "wellbeing*" OR TI "well being*" OR AB "well being*" OR TI "well-being*" OR AB "well-being*" OR MA "Well Being*" OR TI "worry*" OR AB "worry*" OR TI "mood*" OR AB "mood*" AND Limiters: Published Year: 2013-2023; Language: English, |
| **CINAHL** | 167  (27.09.2023) | ((TI "urban*" OR AB "urban*" OR MH "Urban Areas" OR TI "city*" OR AB "city*" OR TI "citie*" OR AB "citie*" OR TI "Cities" OR AB "Cities" OR TI "metropolitan*" OR AB "metropolitan*" OR TI "residential*" OR AB "residential*" OR TI "agglomeration*" OR AB "agglomeration*" OR TI "town*" OR AB "town*" OR TI "built environment*" OR AB "built environment*" OR MH "built environment" OR TI "alley*" OR AB "alley*" OR TI "facade*" OR AB "facade*" OR TI "intersection*" OR AB "intersection*" OR TI "plaza*" OR AB "plaza*" OR TI "road*" OR AB "road*" OR TI "sidewalk*" OR AB "sidewalk*" OR TI "street*" OR AB "street*" OR TI "high building*" OR AB "high building*" OR TI "skyscraper*" OR AB "skyscraper*" OR TI "traffic noise*" OR AB "traffic noise*" OR TI "railway noise*" OR AB "railway noise*" OR TI "highway*" OR AB "highway*" OR TI "forest*" OR AB "forest*" OR TI "greenspace*" OR AB "greenspace*" OR TI "green space*" OR AB "green space*" OR TI "park*" OR AB "park*" OR TI "river*" OR AB "river*" OR TI "tree*" OR AB "tree*" OR TI "trees" OR AB "trees" OR TI "green infrastructure*" OR AB "green infrastructure*" OR TI "vegetation*" OR AB "vegetation*" OR TI "greenway*" OR AB "greenway*" OR TI "bluespace*" OR AB "bluespace*" OR TI "blue space*" OR AB "blue space*" OR TI "vertical green*" OR AB "vertical green*" OR TI "population density*" OR AB "population density*" OR MH "population density" OR TI "peripersonal space*" OR AB "peripersonal space*" OR TI "overcrowding*" OR AB "overcrowding*" OR MH "crowding" OR TI "residential density*" OR AB "residential density*") AND (TI "EEG" OR AB "EEG" OR TI "electroencephalograph*" OR AB "electroencephalograph*" OR MH "electroencephalography" OR TI "meeg*" OR AB "meeg*") AND (TI "mental health*" OR AB "mental health*" OR MH "mental health" OR TI "psychosis" OR AB "psychosis" OR MH "psychotic disorders" OR TI "anxiety*" OR AB "anxiety*" OR MH "Anxiety" OR TI "attention*" OR AB "attention*" OR MH "attention" OR TI "restoration*" OR AB "restoration*" OR TI "calm*" OR AB "calm*" OR TI "cognition*" OR AB "cognition*" OR MH "cognition" OR TI "cognitive function*" OR AB "cognitive function*" OR TI "Depression*" OR AB "Depression*" OR MH "Depression" OR TI "depressive disorder" OR AB "depressive disorder" OR TI "emotional regulation*" OR AB "emotional regulation*" OR MH "Emotional Regulation*" OR TI "fatigue*" OR AB "fatigue*" OR MH "fatigue" OR TI "fear*" OR AB "fear*" OR MH "Fear" OR TI "internalizing disorder*" OR AB "internalizing disorder*" OR TI "relax*" OR AB "relax*" OR MH "Relaxation" OR TI "rest*" OR AB "rest*" OR TI "sleep*" OR AB "sleep*" OR MH "Sleep" OR TI "stress*" OR AB "stress*" OR MH "Stress, Psychological" OR TI "wellbeing*" OR AB "wellbeing*" OR TI "well being*" OR AB "well being*" OR TI "well-being*" OR AB "well-being*" OR MH "Psychological Well-Being*" OR TI "worry*" OR AB "worry*" OR MH "worry" OR TI "mood*" OR AB "mood*" AND Limiters: Published Date: 20130101‐20230927; Language: English, German |
